# A Duplication CNV That Conveys Traits Reciprocal to Metabolic Syndrome and Protects against Diet-Induced Obesity in Mice and Men

**DOI:** 10.1371/journal.pgen.1002713

**Published:** 2012-05-24

**Authors:** Melanie Lacaria, Pradip Saha, Lorraine Potocki, Weimin Bi, Jiong Yan, Santhosh Girirajan, Brooke Burns, Sarah Elsea, Katherina Walz, Lawrence Chan, James R. Lupski, Wenli Gu

**Affiliations:** 1 Department of Molecular and Human Genetics, Baylor College of Medicine, Houston, Texas, United States of America; 2 Diabetes and Endocrinology Research Center, Baylor College of Medicine, Houston, Texas, United States of America; 3 Department of Medicine, Baylor College of Medicine, Houston, Texas, United States of America; 4 Department of Molecular and Cellular Biology, Baylor College of Medicine, Houston, Texas, United States of America; 5 Texas Children's Hospital, Houston, Texas, United States of America; 6 Department of Molecular and Human Genetics, Virginia Commonwealth University, Richmond, Virginia, United States of America; 7 Department of Pediatrics, Virginia Commonwealth University, Richmond, Virginia, United States of America; 8 Department of Human Genetics, University of Miami, Miami, Florida, United States of America; 9 Department of Pediatrics, Baylor College of Medicine, Houston, Texas, United States of America; University of Pennsylvania, United States of America

## Abstract

The functional contribution of CNV to human biology and disease pathophysiology has undergone limited exploration. Recent observations in humans indicate a tentative link between CNV and weight regulation. Smith-Magenis syndrome (SMS), manifesting obesity and hypercholesterolemia, results from a deletion CNV at 17p11.2, but is sometimes due to haploinsufficiency of a single gene, *RAI1*. The reciprocal duplication in 17p11.2 causes Potocki-Lupski syndrome (PTLS). We previously constructed mouse strains with a deletion, *Df(11)17*, or duplication, *Dp(11)17*, of the mouse genomic interval syntenic to the SMS/PTLS region. We demonstrate that *Dp(11)17* is obesity-opposing; it conveys a highly penetrant, strain-independent phenotype of reduced weight, leaner body composition, lower TC/LDL, and increased insulin sensitivity that is not due to alteration in food intake or activity level. When fed with a high-fat diet, *Dp(11)17/+* mice display much less weight gain and metabolic change than WT mice, demonstrating that the *Dp(11)17* CNV protects against metabolic syndrome. Reciprocally, *Df(11)17/+* mice with the deletion CNV have increased weight, higher fat content, decreased HDL, and reduced insulin sensitivity, manifesting a bona fide metabolic syndrome. These observations in the deficiency animal model are supported by human data from 76 SMS subjects. Further, studies on knockout/transgenic mice showed that the metabolic consequences of *Dp(11)17* and *Df(11)17* CNVs are not only due to dosage alterations of *Rai1*, the predominant dosage-sensitive gene for SMS and likely also PTLS. Our experiments in chromosome-engineered mouse CNV models for human genomic disorders demonstrate that a CNV can be causative for weight/metabolic phenotypes. Furthermore, we explored the biology underlying the contribution of CNV to the physiology of weight control and energy metabolism. The high penetrance, strain independence, and resistance to dietary influences associated with the CNVs in this study are features distinct from most SNP–associated metabolic traits and further highlight the potential importance of CNV in the etiology of both obesity and MetS as well as in the protection from these traits.

## Author Summary

Genetic factors play a large role in obesity. However, despite recent technical progress in the search for genetic variants, the identities of causative and contributory genetic factors remain largely unknown. Whereas nucleotide sequence variation has been studied extensively with respect to its potential contribution to obesity, copy number variations (CNV), in which genes exist in abnormal numbers of copies mostly due to duplication or deletion, have only more recently been observed to be associated with human obesity. In this report, we utilize chromosome engineered mouse strains harboring a deletion or duplication CNV to address the potential functional impact of CNVs on weight control and metabolism. We show that the duplication CNV leads to lower body weight; it is also metabolically advantageous and protects from diet-induced obesity and metabolic syndrome (MetS). The deletion CNV causes a “mirror” phenotype with increased body weight and MetS–like phenotypes. Importantly, these effects manifest regardless of the genetic background and do not appear to be attributable to any single gene. These findings demonstrate experimentally that CNV can be causative for weight and metabolic phenotypes and highlight the potential relevance and importance of CNV in the etiology of obesity/MetS and the protection from these traits.

## Introduction

The significance of copy number variation (CNV) in human genetic variation is now indisputable [1], [2]. However, in contrast to the revolutionary progress achieved in the discovery of CNVs and delineating the mechanisms for their formation, our current knowledge of the downstream functional mechanisms by which CNVs contribute to trait manifestations is limited. Functional contributions of CNV to human biology have only been examined in a few physiological systems including the neuropsychiatric/behavioral fields [2], [3].

About 400 million people worldwide are classified as obese [4] and are likely to suffer from premature mortality and obesity-associated morbidities, such as hyperglycemia, dyslipidemia, hypertension and metabolic syndrome (MetS) [5]. The etiologies for obesity include genetic contributions [4], but the identities of the specific genetic factors remain largely unknown. Single nucleotide polymorphisms (SNPs) identified through linkage and genome-wide association studies (GWAS) explain only 1–2% of the variation in obesity phenotypes as measured by BMI [6], [7], [8].

Recent observations in humans indicate a tentative link between CNV and weight regulation. Deletions at 16p11.2 were associated with a highly penetrant form of obesity often found with hyperphagia and intellectual disabilities, whereas the reciprocal duplication conveys a 8.3 fold increased risk for being clinically underweight [9], [10]. These comprehensive studies on patients added to the clinical observations of obesity associated with CNV that have been noted for several chromosomal syndromes and genomic disorders including Down [11] and Prader-Willi syndromes [12]. However, there is no experimental data that proves the causative role of the CNV in the abnormality in weight regulation, nor is there any study on the biology underlying this tentative link.

Potocki-Lupski syndrome (PTLS, MIM 610883) [13], [14] is an intellectual disability and multiple congenital anomalies (ID/MCA) syndrome due to a heterozygous interstitial duplication CNV in chromosome 17p11.2. Mildly lowered total cholesterol and LDL were noted for some PTLS patients [13]. The reciprocal deletion CNV of the same interval causes a distinct ID/MCA disorder known as Smith-Magenis syndrome (SMS, MIM 182290) [15], [16], [17]. Obesity and hypercholesterolemia are phenotypes of SMS [16], [18], [19]. By chromosomal engineering, we previously constructed mouse models of PTLS and SMS carrying the duplication [*Dp(11)17*] or deletion [*Df(11)17*] of a 2 Mb chromosomal segment that includes the majority of the mouse region syntenic to the PTLS/SMS common recurrent CNV interval (Figure S1) [20]. Both *Dp(11)17/+* and *Df(11)17/+* mice partially recapitulate the respective human phenotypes such as craniofacial abnormalities [21], [22], altered learning, memory and social interaction [20], [23], [24], [25], and display a transcriptome [25] that is distinct from their wild type (WT) littermates.

In the context of exploring the biological link between CNV and weight control, we now utilize these mouse models to investigate the detailed metabolic consequences of the PTLS duplication CNV and the reciprocal SMS deletion CNV. To simplify data analyses, all experiments were performed with male animals.

## Results

### Phenotypes reciprocal to metabolic syndrome in *Dp(11)17* animals

**Figure 1:**
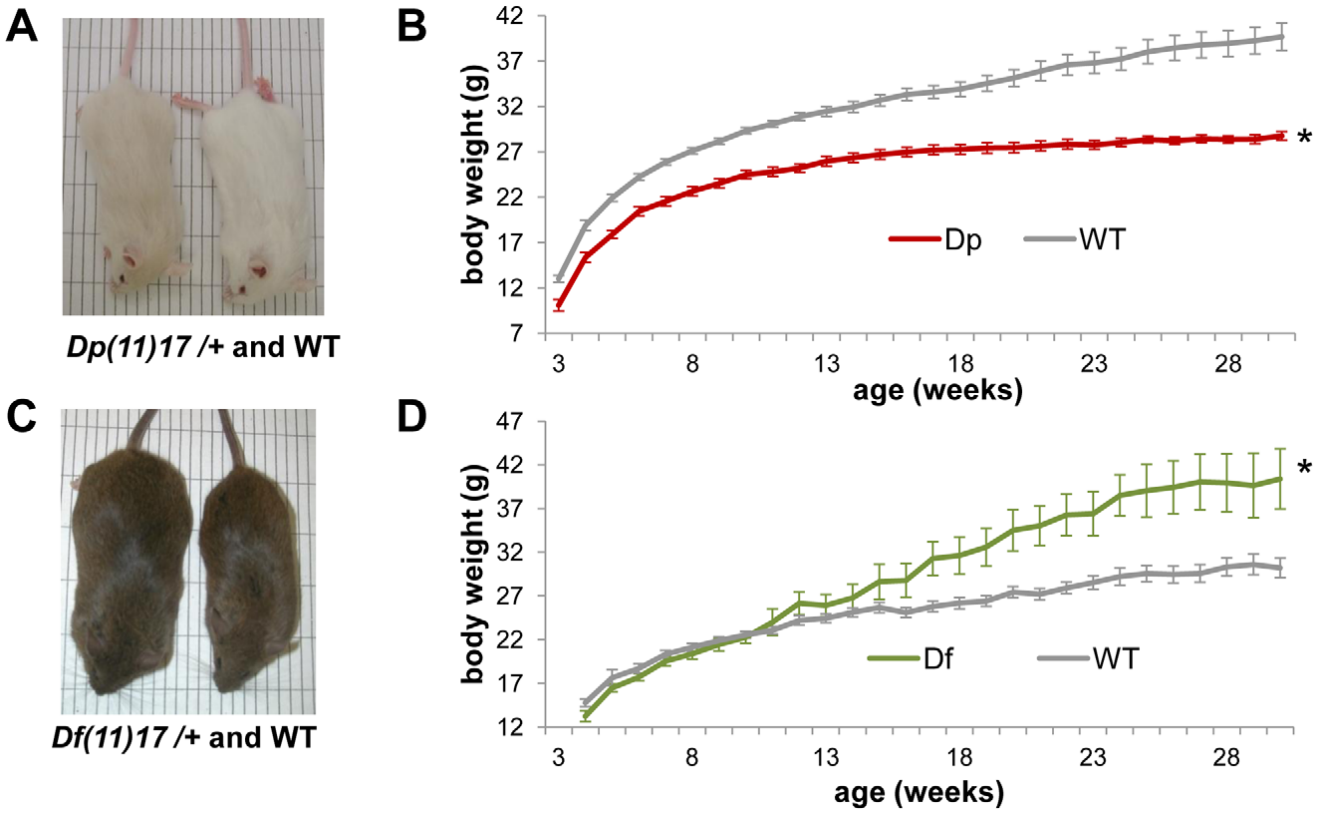
*Dp(11)17/+* mice have reduced and *Df(11)17/+* mice have increased body weight. (A) A *Dp(11)17/+* male (23 weeks old) and its WT littermate are shown, both on isogenic C57BL/6*^Tyr^* ^c*-Brd*^ background. *Dp(11)17/+* mice appear gray because of a coat color marker in the construct used to chromosome engineer this strain [20]. (B) Growth curve of *Dp(11)17/+* (red) and WT littermates (gray) reveal decreased weights for the duplication CNV mutants throughout their life span (*p<0.001 for by ANOVA with repeated measures). (C) A *Df(11)17/+* male (32 weeks old) and its WT littermate on pure 129S5 background (D) Growth curve of *Df(11)17/+* (green) and WT littermates (gray) reveal increased weights for the deletion CNV mutants (*p<0.05 by ANOVA). (B, D): n = 10–25 mice for each data point, results are expressed as mean ± s.e.m.

First, we found that, similar to previous observations on several genetic backgrounds (C57BL/6J/129S5 and N7 or N12 congenic C57BL/6J) [20], [25], [26], the *Dp(11)17/+* mice on an isogenic (N>17) C57BL/6J background also display significantly reduced body weight compared to their WT littermates (Figure 1A, 1B). In contrast, and again in accordance with earlier reports on different backgrounds (C57BL/6J/129S5 and N7 or N12 congenic C57BL/6J) [20], [25], the *Df(11)17/+* mice with the reciprocal deletion CNV on a pure (N>10) 129S5 background are significantly heavier than their WT littermates after 15 weeks of age (Figure 1C, 1D). Thus, the reciprocal duplication and deletion CNVs not only change body weight in opposing directions, but also, strikingly, do so in a highly penetrant manner that is independent of the genetic background. Highly penetrant weight change phenotypes were recently also observed in humans for two obesity-associated deletion CNVs on 16p11.2 and the reciprocal duplication of one of them associated with leanness [9], [10], [27]. The high penetrance differentiates CNV-associated obesity from SNP associated obesity in which, except for some very rare mutations in a few genes of the leptin/melanocortin pathway (*LEP*, *MC4R*, etc.), almost all variants have low penetrance [28], [29].

**Figure 2:**
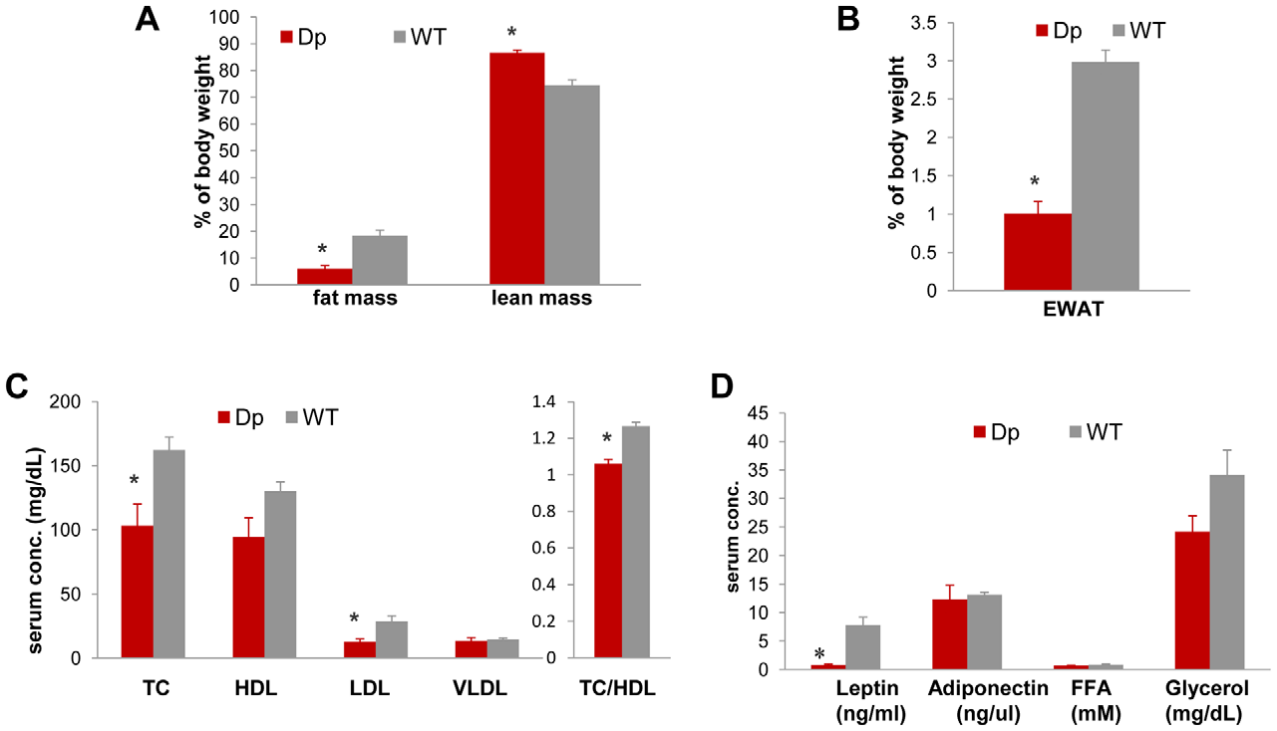
*Dp(11)17/+* mice (red) are also leaner and have reduced serum TC, LDL, TC/HDL ratio, and leptin. (A) Less relative total fat mass (*p = 0.000088) and more relative lean mass (*p = 0.00012) was identified in *Dp(11)17/+* mice with ECHO-MRI system. (B) *Dp(11)17/+* animals also possess smaller epididymal white adipose tissue pad (EWAT) (*p = 0.0020). Fasting serum profile revealed (C) reduced TC (*p = 0.021), LDL (*p = 0.01), TC/HDL ratio (*p = 0.0007) and (D) reduced leptin (*p = 0.021) in *Dp(11)17/+* mice. All comparisons were made with two-tailed t-test; results are expressed as mean ± s.e.m. from measurements of (A) 6 *Dp(11)17/+* and 10 WT at 21–22 wks (B) 6 *Dp(11)17/+*, 7 WT at 41 wks (C) 5 *Dp(11)17/+* and 6 WT at 20–22 wks (D) 6 *Dp(11)17/+* and 4 WT of 20–21 wks.

In addition to having reduced weight, adult *Dp(11)17/+* mice on an isogenic (N>17) C57BL/6J background are also leaner than WT males, as measured via ECHO-MRI whole body scans (Echo medical systems, Texas) and manual dissection. *Dp(11)17/+* mice have a significantly lower percentage of both whole body fat mass and epididymal white adipose tissue (EWAT) (Figure 2A, 2B), as well as a significantly higher percentage of lean mass (Figure 2A). These findings are in accordance with our previous reports of reduced abdominal fat in *Dp(11)17/+* mice on different strain backgrounds (N7 [26] and N12 C57BL/6J [25]), further demonstrating the strain-independent manifestation of the metabolic phenotypes caused by the duplication CNV. Moreover, adult *Dp(11)17/+* mice display significantly reduced fasting total serum cholesterol (TC) and LDL levels (Figure 2C) as well as a cardioprotective decrease of TC/HDL ratio (Figure 2C). Interestingly, the change in TC and LDL resembles the clinical observations in PTLS patients [13], despite the mechanistic differences in lipid metabolism between human and mouse [30]. Consistent with their lower adiposity [31], the serum leptin concentration is decreased in *Dp(11)17/+* mice (Figure 2D).

**Figure 3:**
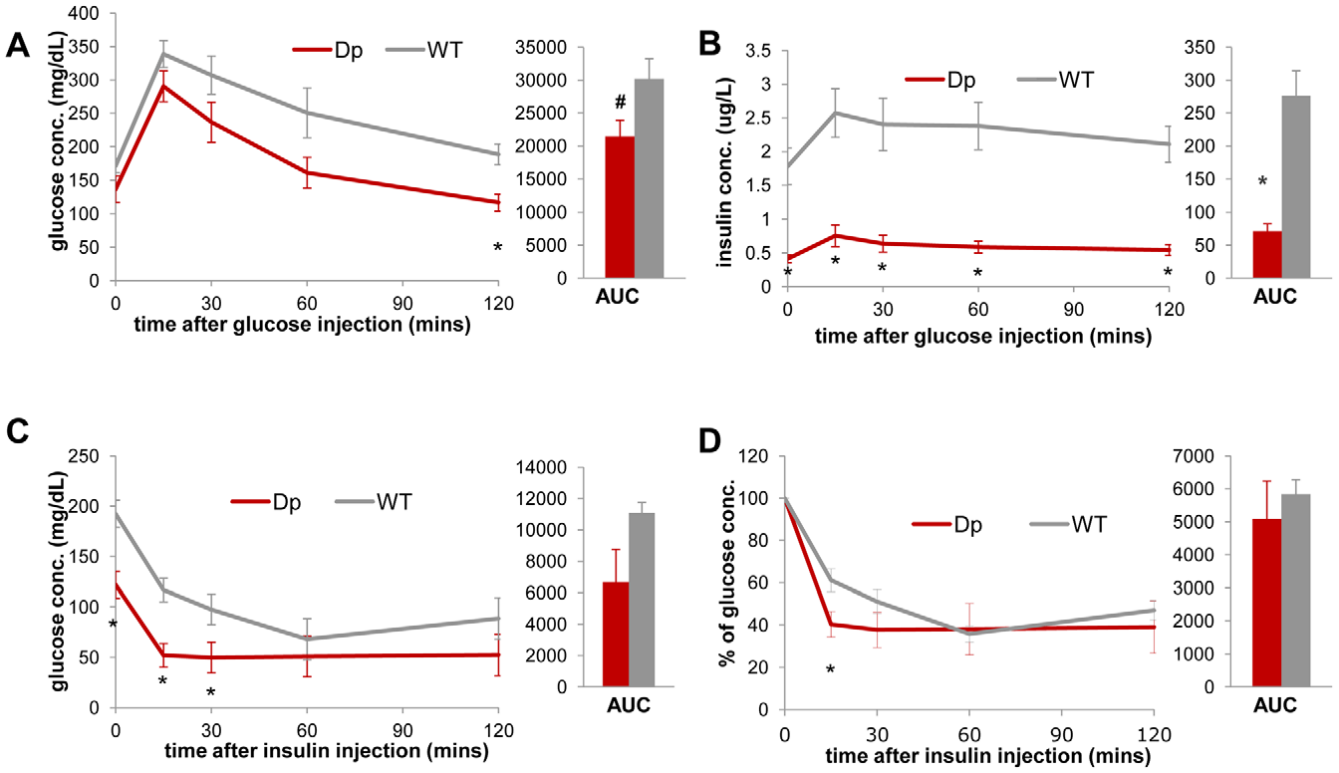
*Dp(11)17/+* mice (red) display improved insulin sensitivity compared to WT mice (gray). During IP-GTT (6 hr fasting, 1.5 mg glucose/g body weight), *Dp(11)17/+* mice demonstrate (A) lower blood glucose (*p = 0.006 for 120 minutes post injection; # p = 0.052 for the area under curve (AUC)) and (B) lower blood insulin level (*p = 0.0037, 0.0026, 0.0051, 0.0031 and 0.0015 for the time points 0, 15, 30, 60 and 120 minutes; *p = 0.002 for AUC). During IP-ITT (4–6 hrs fasting, 1 mU insulin/g body weight), *Dp(11)17/+* mice also demonstrate lower blood glucose concentration, shown as both actual concentration (C) (*p = 0.011, 0.004 and 0.037 for 0, 15 and 30 mins post insulin injection) and percentage of the initial glucose concentration (D) (*p = 0.038 for 15 mins post insulin injection). All comparisons were made with two-tailed t-test; results are expressed as mean ± s.e.m. from measurements of (A, B) n = 5 *Dp(11)17/+* and 6 WT at 30 wks (C, D) 4 *Dp(11)17/+* and 4 WT mice at 20–22 wks. All AUCs are computed until 120 minutes, for the entire length of the time curves.

Furthermore, the intraperitoneal glucose tolerance test (IP-GTT) demonstrates an overall improved glucose clearance in *Dp(11)17/+* mice compared to WT littermates; the difference in their serum glucose concentration becomes significant at 120 minutes (Figure 3A). The plasma insulin levels during the GTT were significantly lower in the *Dp(11)17/+* animals throughout the test, suggesting that the improvement in glucose tolerance was not due to increased insulin production by the pancreas, but likely the result of improved insulin sensitivity (Figure 3B). Indeed, in the insulin tolerance test (ITT), insulin injection lowered blood glucose levels significantly faster in *Dp(11)17/+* than in WT mice, further corroborating their increased insulin sensitivity (Figure 3C and 3D). Intriguingly, the circulating concentration of adiponectin is not changed in *Dp(11)17/+* mice (Figure 2D), suggesting that adiponectin-independent pathways are involved in the alteration of their insulin sensitivity [32].

### The *Dp(11)17* CNV results in higher intrinsic metabolic activity

**Figure 4:**
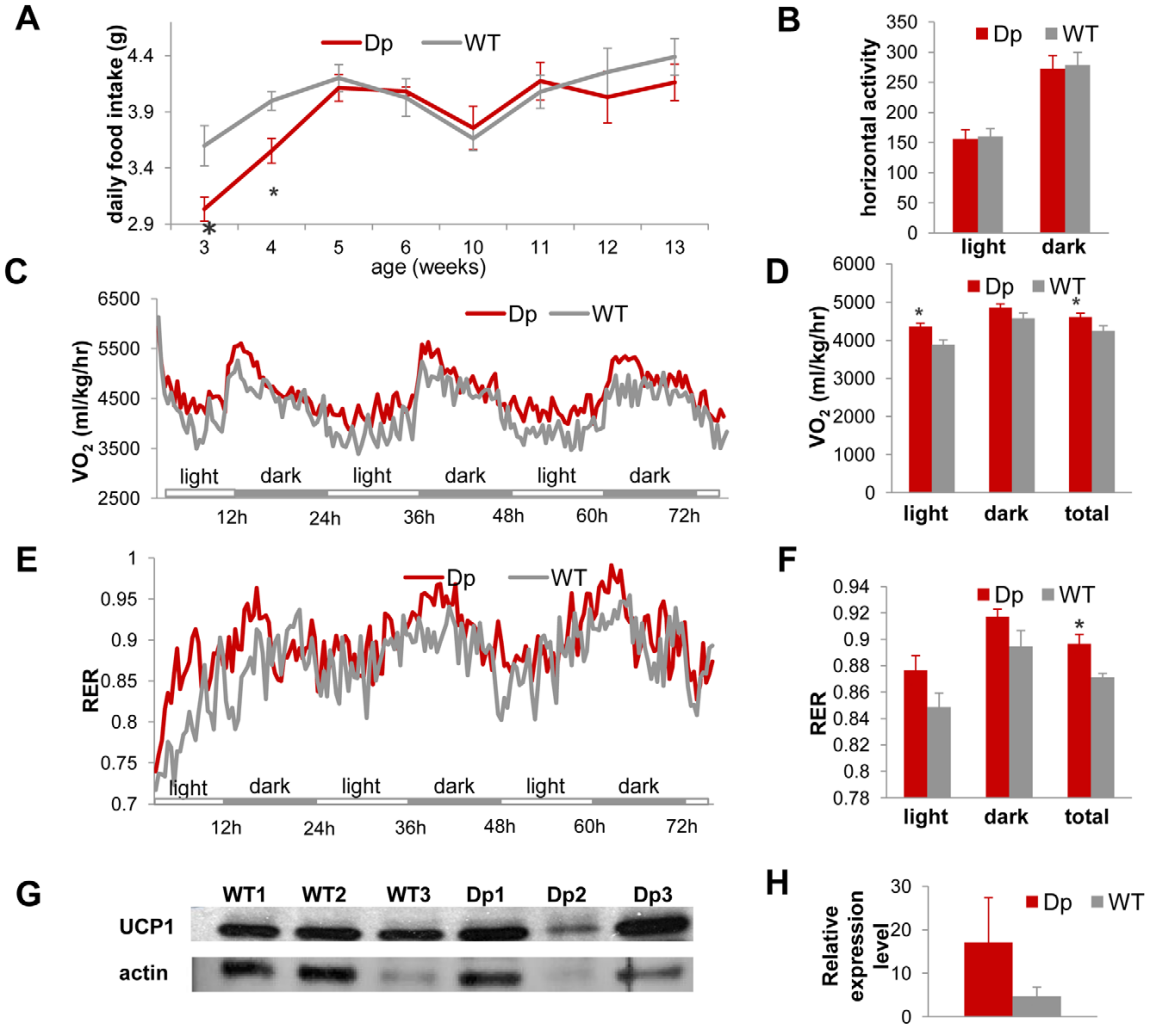
*Dp(11)17/+* mice (red) have similar food intake and activity levels, but higher energy expenditure than WT mice (gray), which may be partially accounted for by the difference in expression levels of UCP1 in the BAT tissue. (A) *Dp(11)17/+* mice have similar amount daily food intake to WT mice after 4 wks of age, although they consume less food at 3 wks (*p = 0.001) and 4 wks (*p = 0.048). (B) VersaMax system (Accuscan Inc., Ohio) using the beam block technique implemented in home cages revealed no difference in horizontal activity level between *Dp(11)17/+* and WT animals. (C, D) Oxygen consumption measured using the CLAMS system (Columbus Ins., Ohio) for over three days documented higher energy expenditure of *Dp(11)17/+* mice in the light phases alone (*p = 0.0095) and during the entire day (*p = 0.044). (E, F) Respiratory exchange ratio (RER) measured using the CLAMS system for over three days again confirmed higher metabolic activity of *Dp(11)17/+* mice (*p = 0.00151). (G) Western blot for UCP1 expression in BAT tissue of three *Dp(11)17/+* and three WT mice with antibody AB3036 (Millipore). The same blot was normalized to actin blotting using MAB1501 (Millipore). (H) Normalized intensity of UCP1 signals in *Dp(11)17/+* vs. WT mice (17.13±10.21 vs. 4.74±2.05, p = 0.35). The measurements are from (A) 5–13 *Dp(11)17*/+ and 5–11 WT at different time points (B) to (F) 12 *Dp(11)17*/+ and 7–10 WT at 25–32 wks (G) 3 *Dp(11)17*/+ and 3 WT at 30 wks.

We found that the reduction in body weight of *Dp(11)17/+* mice is not simply due to an altered energy intake or increased activity since they consume identical amounts of food after four weeks of age (Figure 4A) and have similar activity levels to those of their WT littermates (Figure 4B). Thus, intrinsic changes in energy expenditure likely explain the observed phenotypes. Indeed, as assayed by indirect calorimetry, *Dp(11)17/+* mice demonstrate an overall higher oxygen consumption (VO_2_) per lean mass and higher respiratory exchange ratio (RER) than WT mice, indicating higher energy expenditure than their WT littermates (Figure 4C–4F). Western blotting suggested an elevated expression level of protein uncoupled 1 (UCP1) in brown adipose tissue (BAT) of the *Dp(11)17/+* mice (Figure 4G, 4H), although there is considerable variability in UCP1 expression among different samples. UCP1 is a key component of thermogenesis in BAT [33], this difference may partially explain the higher intrinsic energy expenditure of the *Dp(11)17/+* mice. Interestingly, *Dp(11)17/+* mice also appear to have a trend toward slightly higher body temperature (34.56±0.34°C) than WT littermates (33.9±0.90°C). We did not observe differences in the expression levels of UCP2 and UCP3 in BAT between *Dp(11)17/+* and WT mice, nor did we observe differences in other signature metabolism genes (*Glut4*, *AP2* in BAT, and *Lpk*, *Fas*, *Acc1*, *Srebp1c*, *Tnxip* in the liver) (Figure S4A, S4B).

Thus, while on a regular chow diet, *Dp(11)17/+* mice are leaner than their WT littermates, and they have lower serum TC/LDL levels and reduced leptin concentration. *Dp(11)17/+* mice also appear to be more insulin sensitive and have higher energy expenditure but display no difference in activity level in comparison to WT mice. These traits are reciprocal or antithetical to those of metabolic syndrome and most appear to manifest independent of the genetic background of the mouse strain.

Importantly, except for the parameters related to energy metabolism, a comprehensive serum analysis did not observe any difference in other serum chemistry parameters between *Dp(11)17/+* and WT animals (Table S1). Also, under daily evaluation by veterinarian staff in our mouse facility, no overt illness was observed in *Dp(11)17/+* mice. Combined with the fact that *Dp(11)17/+* animals also have identical activity level and food intake to WT mice, the metabolic traits we observed in *Dp(11)17/+* mice are unlikely due to any illness related to the duplication CNV, but rather a direct effect from the CNV.

### The *Dp(11)17* CNV protects against diet-induced obesity (DIO)

**Figure 5:**
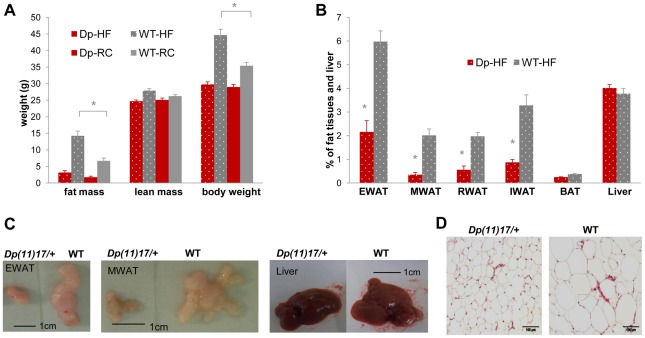
*Dp(11)17/+* mice (red) display resistance to diet-induced obesity compared to WT littermates (gray) after a high-fat diet (HF) feeding from 19 to 22 weeks. (A) Only WT, but not *Dp(11)17/+* mice have significant (*p = 0.00028) weight gain after three weeks of HF (19–22 wks) that is mainly due to fat mass increase (*p = 0.00030). (B) Body weight percentage of epididymal (EWAT), mesenteric (MWAT), retroperitoneal (RWAT) and inguinal (IWAT) white adipose tissues are all higher in WT mice post HF than *Dp(11)17/+* mice (*p = 0.000033 for EWAT, p = 0.00021 for MWAT, p = 0.000033 for RWAT, p = 0.000048 for IWAT). Liver and brown adipose tissues (BAT) remain similar. (C) Dissected EWAT, MWAT and liver are compared between WT and *Dp(11)17/+* mice post HF diet. EWAT and MWAT, but not the liver, are much smaller in *Dp(11)17/+* mice. (D) Histology of the EWAT adipocytes from *Dp(11)17/+* and WT mice, demonstrating smaller adipocytes in *Dp(11)17/+* mice after HF feeding. The measurements are from (A): 11 *Dp(11)17*/+ and 12 WT at 22 wks post HF feeding compared to 6 *Dp(11)17*/+ and 10 WT on RC at 21–22 wks (B) 8 *Dp(11)17*/+ and 9 WT post HF feeding. *Dp*/WT mice after HF diet: red/gray bars with dotted pattern; *Dp*/WT mice with RC: red/gray bars without pattern.

**Figure 6:**
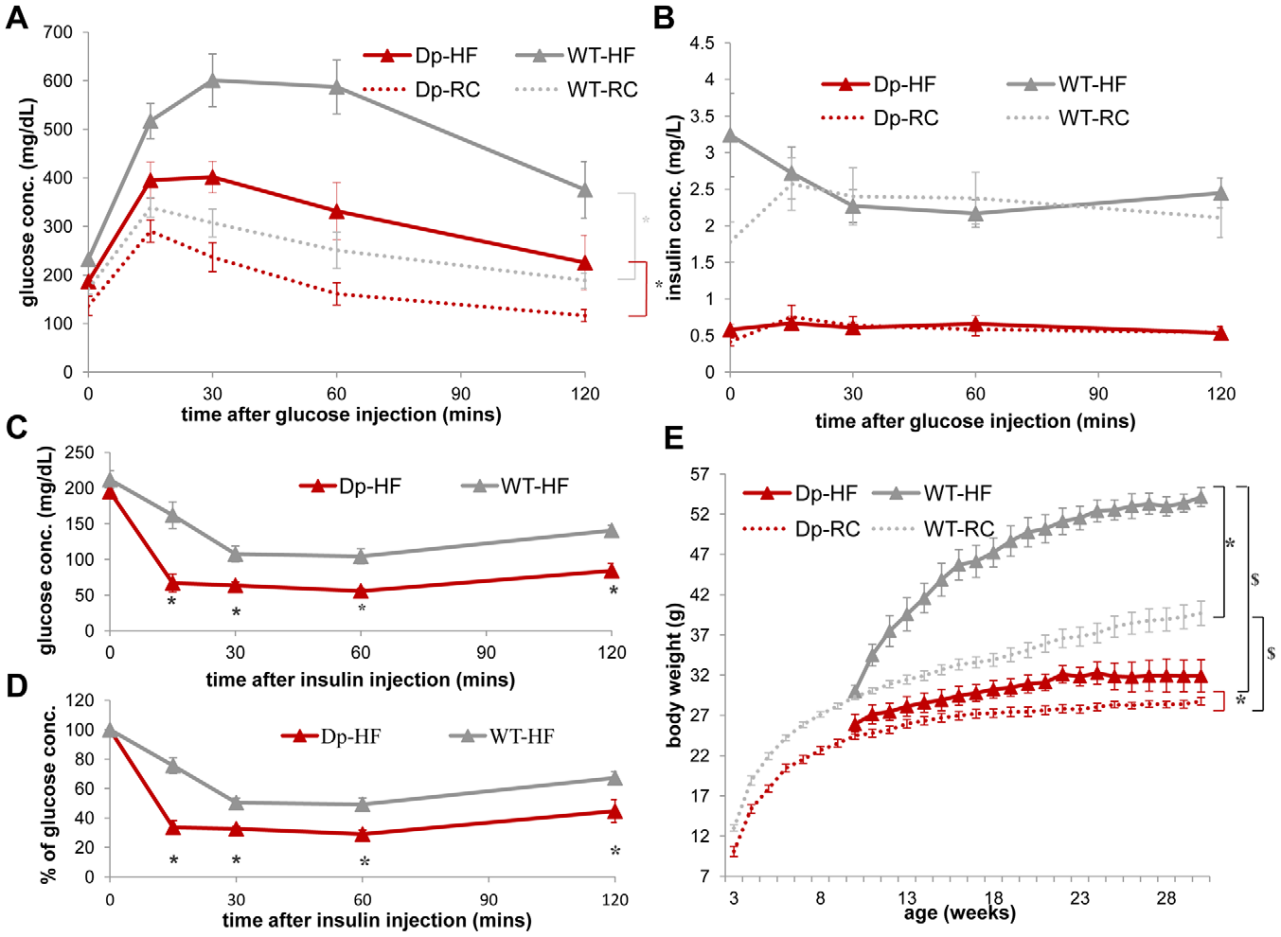
WT mice (gray) display a greater increase in insulin resistance than *Dp(11)17/+* mice (red) after HF diet from 19 to 22 weeks as well as an increased weight gain after a long-term HF diet. (A) During IP-GTT, WT mice display more dramatically decreased glucose clearance rate (*p = 0.00011) after HF diet than *Dp(11)17/+* mice (*p = 0.03). (B) The insulin level of both genotypes are not impacted by HF diet. (C, D) During IP-ITT, *Dp(11)17/+* mice after HF diet demonstrate lower blood glucose concentration than WT mice, shown as both actual concentration in (C) (*p = 0.002, 0.007, 0.003 and 0.002) and percentage of the initial glucose concentration in (D) (*p = 0.00015, 0.00063, 0.0026, 0.041); whereas the differences are only partially significant under RC as shown in Figure 3 (C, D). (E) Body weight of both genotypes after HF diet from 10 to 30 weeks. *: comparison for each genotype between HF and RC; $: comparison between the genotypes under the same diet condition. After HF feeding, both genotypes gain weight (WT: *p<0.001; *Dp(11)17/+*: *p = 0.048). *Dp(11)17/+* mice are still lighter than WT littermates after HF (^$^p<0.0005), similar to those fed with RC (^$^p<0.001). The curves for normal diet (data points without triangles) are the same as shown in Figure 1B. All comparisons were made with ANOVA with repeated measures (A, B, E) or two-tailed t-test (C, D); results are expressed as mean ± s.e.m. and obtained from measurements of n = 5 *Dp(11)17/+* and 7 WT after HF diet. *Dp*/WT mice after HF diet: red/gray solid line with triangle markers; *Dp*/WT mice with RC: red/gray dashed line without marker.

Next, we investigated potential influences of the *Dp(11)17* CNV on the genetic susceptibility to diet induced obesity (DIO). First, we placed *Dp(11)17/+* and WT mice on a HF (60% calories from fat) diet for three weeks (19 to 22) after 19 weeks of a normal diet. After these three weeks, the WT mice had dramatically increased body weight compared to control WT mice of the same age that have been kept on a regular chow (RC) diet. The *Dp(11)17/+* mice, however, did not display significant weight gain compared to other *Dp(11)17/+* animals on RC (Figure 5A). More specifically, the weight gain in WT mice is predominantly due to an increase in the amount of fat mass that is only observed in WT and not *Dp(11)17/+* mice (Figure 5A). Indeed, while BAT and liver remain of similar sizes (relative to body weight) between the two genotypes after HF diet, the WAT tissues (from four body locations: epididymal, mesenteric, retroperitoneal and inguinal WAT) are much smaller in *Dp(11)17/+* than WT mice (Figure 5B, 5C), accompanied by smaller-sized adipocytes (Figure 5D). Furthermore, for WT mice, HF diet resulted in a marked decrease in glucose clearance during GTT but no change in blood insulin level; whereas the glucose clearance in *Dp(11)17/+* mice is much less affected by the HF diet (Figure 6A, 6B). This difference in the extent of HF diet mediated insulin resistance between WT and *Dp(11)17/+* mice was further confirmed in ITT experiments, which demonstrate even more significant differences between the two genotypes after HF diet (Figure 6C, 6D).

Next, to explore the long-term impact of the *Dp(11)17* CNV in DIO, we examined *Dp(11)17/+* males along with their WT littermates on a 42% fat HF diet starting from week 3 for 20 weeks. While the high-fat diet causes massive weight gain in WT mice and literally “supersizes” these animals, it produces only a minimal increase in body weight in the *Dp(11)17/+* mice, confirming the salient resistance of the *Dp(11)17/+* genotype to diet-induced weight gain (Figure 6E). In aggregate, these findings demonstrate the salubrious effect of this duplication CNV in that it provides protection against diet-induced obesity and insulin resistance.

### *Rai1* gain alone is insufficient to account for CNV–associated metabolic derangements

We next examined whether the metabolic traits conveyed by the duplication CNV are due to the copy number gain of a single gene. The typical CNV interval of PTLS/SMS encompasses over 40 human genes; one of them, *retinoic acid induced 1* (*RAI1*), is considered the “predominant” causative gene in the deletion CNV interval mediating the majority of SMS clinical findings through haploinsufficiency [15], [16], [17]. Also, for PTLS, *RAI1* is a major dosage sensitive gene contributing to the phenotype, as suggested by duplication mapping in humans [34] and the rescue of selected phenotypes after normalizing the gene dosage of *Rai1* to n = 2 in *Dp(11)17/Rai1^−^* animals [26]. To examine the contribution of the *RAI1/Rai1* gene to the metabolic phenotypes of PTLS, we compared the metabolic profile of *TgRai1* animals [35] that overexpress *Rai1* but do not have copy number change of most of the surrounding genomic regions to that of *Dp(11)17/+* mice. Although the regulation of *Rai1* expression in *TgRai1* mice is mechanistically different from that in *Dp(11)17/+* mice, in which *Rai1* is localized in a large genomic segment that has a well-defined duplication of the genome, expression studies demonstrated that the *Rai1* “steady state” expression level is similar in *TgRai1* [35] and *Dp(11)17/+* mice [25] (1.5 fold that of WT). Interestingly, *TgRai1* animals display an initial growth retardation; however, they eventually normalize their body weight by 20 weeks of age [35]. This is distinct from the *Dp(11)17/+* mice, whose difference in body weight when compared to their WT littermates remains and even exacerbates as they age (Figure 1). Further, *TgRai1* animals do not demonstrate the dramatically altered body composition and serum chemistry displayed by *Dp(11)17/+* mice (Figure S2A, S2B). Finally, again in striking contrast to the remarkably improved insulin sensitivity and glucose clearance of *Dp(11)17/+* mice, *TgRai1* animals demonstrate no significant differences in their blood glucose or plasma insulin during GTT when compared with WTs (Figure S2C, S2D). We conclude that the dosage or steady state expression level of *RAI1/Rai1* is unlikely the sole or major contributor to the obesity opposing and protective metabolic phenotypes observed in the PTLS mice.

### The *Df(11)17* deletion CNV conveys metabolic syndrome like phenotypes in both mouse and human

**Figure 7:**
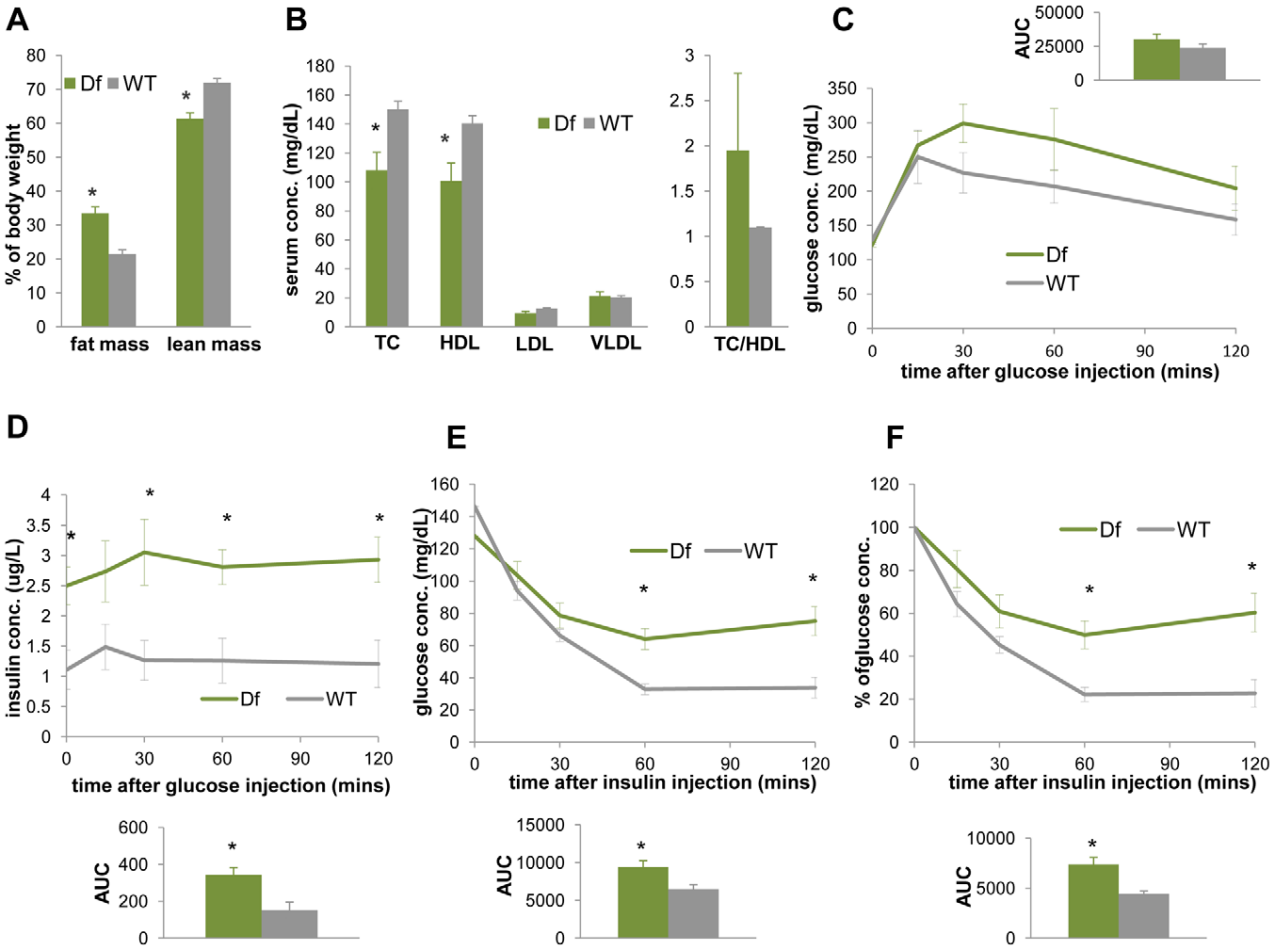
*Df(11)17/+* mice (green) are obese, have reduced TC, HDL, and display reduced insulin sensitivity in comparison to WT mice (gray). (A) ECHO-MRI identified elevated fat mass (*p = 0.0041) and reduced lean mass (*p = 0.0034) in *Df(11)17/+* animals. (B) *Df(11)17/+* animals have lower serum TC (*p = 0.033) and lower HDL (*p = 0.039), but no significantly change in TC/HDL ratio. IP-GTT documented (C) similar blood glucose levels but (D) significantly higher insulin levels (*p = 0.015, 0.028, 0.012 and 0.013 at 0, 30, 60, 120 mins post injection and *p = 0.011 for AUC) in *Df(11)17/+* animals. During IP-ITT, *Df(11)17/+* mice retain higher blood glucose concentration, shown as both (E) actual concentration (*p = 0.026 and 0.008 for 60 and 120 mins post insulin injection and *p = 0.018 for AUC) and (F) percentage of the initial glucose concentration (*p = 0.01 for both 60 and 120 min after injection and *p = 0.0086 for AUC). All comparisons were made with two-tailed t-test; results are expressed as mean ± s.e.m. from measurements of (A) 5 *Df(11)17/+* and 7 WT mice at 32–36 wks (B) 6 *Df(11)17/+* and 6 WT mice at 34–37 wks (C, D) 5 *Df(11)17/+* and 5 WT mice at 37–41 wks (E, F) 5 *Df(11)17/+* and 6 WT mice at 33–37 wks. All AUCs are computed until 120 minutes, for the entire length of the time curves.

After studying the *Dp(11)17/+* mice, we sought to characterize the metabolic profile of the *Df(11)17/+* deletion mice on a fully congenic (N>10) 129S5 background. In mirror image contrast to the observations in *Dp(11)17/+* mice, *Df(11)17/+* mice have not only increased body weight, but also significantly increased percentage of body fat and decreased percentage of lean mass (Figure 7A). These findings are again in accordance with our previous reports of increased size of abdominal fat pad in *Df(11)17/+* mice on mixed [20] and congenic (N>12) C57BL/6J [25] strain backgrounds. Intriguingly, *Df(11)17/+* mice also have reduced TC, similar to *Dp(11)17/+* mice (Figure 7B). However, in contrast to the reduction of the atherogenic LDL in the case of *Dp(11)17/+* animals, the reduced TC of *Df(11)17/+* mice is the result of a reduced HDL, a cardioprotective species of plasma lipoprotein (Figure 7B). The TC/HDL ratio appears higher in *Df(11)17/+* mice although the difference is not significant (Figure 7B). During the GTT, *Df(11)17/+* mice display an impaired glucose tolerance phenotype (Figure 7C) accompanied by significantly higher plasma insulin levels during the test as compared with WT mice (Figure 7D), suggesting that *Df(11)17/+* mice indeed have increased insulin resistance. This interpretation is bolstered by a blunted blood glucose decrement in response to insulin injection during an insulin tolerance test (ITT) in *Df(11)17/+* mice as compared to WT mice (Figure 7E and 7F). The increased insulin resistance and impaired glucose tolerance in *Df(11)17/+* mice, along with the increased body weight, relative adiposity and reduced HDL further document that, metabolically, *Df(11)17/+* mice display endophenotypes that resemble a *bona fide* metabolic syndrome. We did not find significant differences in the level of UCP1 protein between *Df(11)17/+* and WT mice (Figure S4C, S4D).

From studies of human patients, a meta-analysis of 105 cases [16] including both children and adults concluded that 33.3% of the SMS patients are overweight (BMI>24). In a study of 49 SMS children (0.6 to 17.6 years), Smith *et al.* [19] observed that SMS boys had a significantly higher BMI than the published age-matched standards. To systematically address the potential obesity directly caused by the SMS deletion CNV in the context of the population norm, we compared 179 height and 216 weight measurements from 76 subjects with SMS aged between newborn and 46 years (Figure S3) to the population mean of the same age/gender from the center for disease control and prevention (http://www.cdc.gov/growthcharts/). We found that both male and female SMS individuals of all age categories in this cohort are shorter than the general population (Figure S3A, S3D, S3G). Male SMS subjects do not have significant weight abnormalities (Figure S3B, S3H). Females below 11 years have weights below the population mean, whereas those older than 12 years appear heavier than the population mean, although the difference is not significant (Figure S3E, S3H). Most importantly, male SMS subjects older than 2 years and female subjects older than 20 years have significantly higher BMI values than the general population (Figure S3C, S3F, S3I). These observations are consistent with the interpretation that the SMS deletion CNV indeed causes a higher BMI in humans and thus conveys an increased risk for obesity. Smith *et al* [19] also found that the mean fasting TC of SMS patients in their cohort was significantly higher than published pediatric age-matched norms. The aggregate of weight gain and elevated TC in human SMS patients together with the weight gain and insulin resistance in the *Df(11)17/+* mice is consistent with MetS-like traits as part of the SMS endophenotypes.

### *Rai1* haploinsufficiency partially contributes to the *Df(11)17*-mediated phenotype

**Figure 8:**
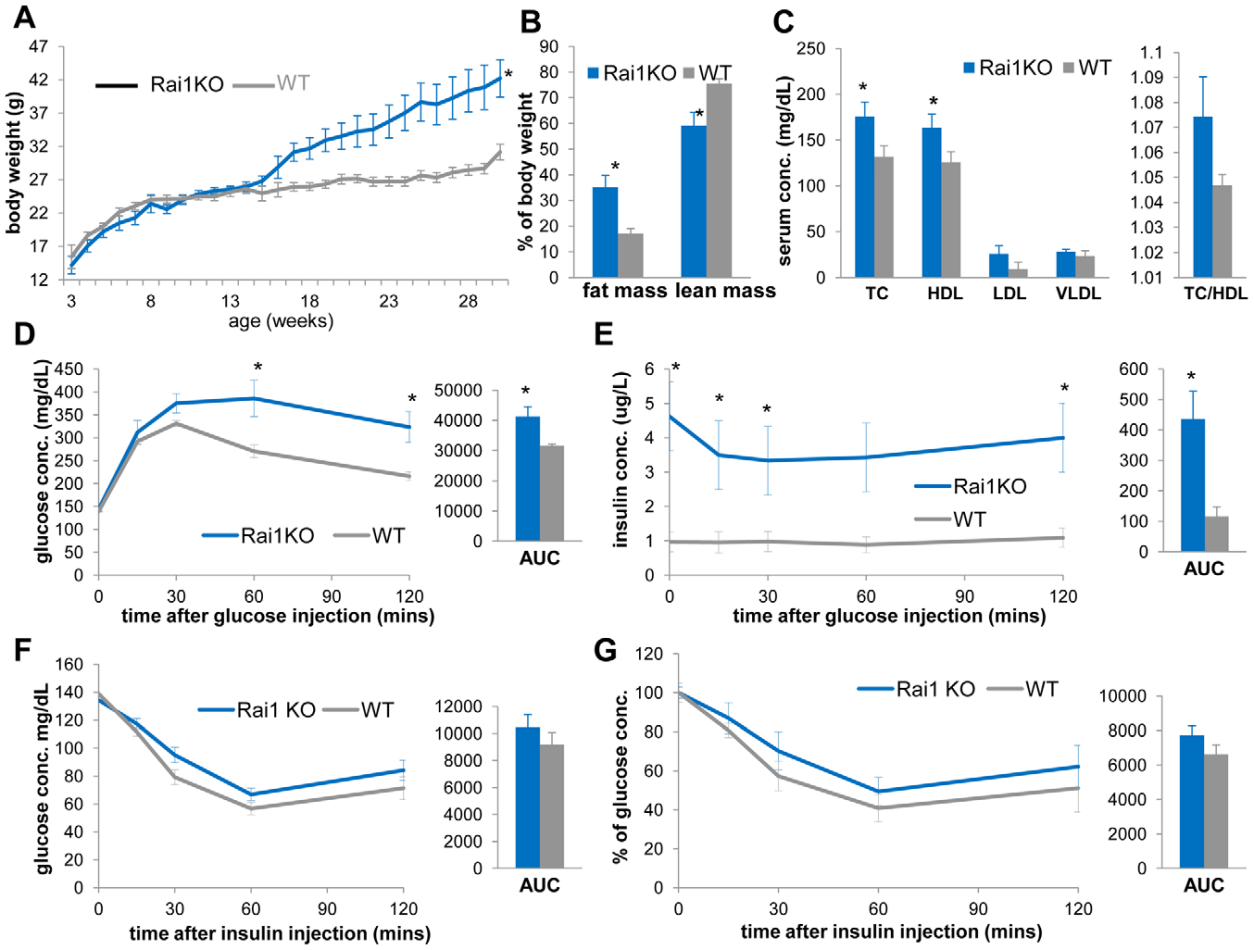
*Rai1^+/−^* mice (blue) are obese, have increased TC, HDL, and display reduced insulin sensitivity compared to WT mice (gray). (A) The growth curve of *Rai1^+/−^* and WT littermates reveals increased body weights of *Rai1^+/−^* mice (*p = 0.028 by ANOVA with repeated measures). (B) *Rai1^+/−^* mice have increased body fat mass (*p = 0.014) and reduced lean mass (*p = 0.015). (C) *Rai1^+/−^* mice demonstrate higher TC (*p = 0.036) and HDL (*p = 0.048), but unchanged TC/HDL ratio. In GTT experiments, *Rai1^+/−^* mice have (D) higher blood glucose at 60 mins (*p = 0.042) and 120 mins (*p = 0.030 after glucose injection, and *p = 0.038 for total AUC) and (E) higher insulin levels throughout (*p = 0.0056, 0.0069, 0.029, 0.008 at 0, 15, 30 and 120 mins and #p = 0.058 at 60 mins after injection. For AUC, *p = 0.021). (F, G) IP-ITT resulted in similar glucose level change between *Rai1^+/−^* and WT mice, as shown by both actual concentration (F) and percentage of the initial glucose concentration (G). All comparisons were made with two-tailed t-test except (A) that used ANOVA; results are expressed as mean ± s.e.m. from measurements of (A) 10–25 *Df(11)17/+* and 10–25 WT mice (B, C) 6 *Rai1^+/−^* and 8 WT at 30–31 wks (D, E) 5 *Rai1^+/−^* and 5 WT mice at 41–43 wks (F, G) n = 7 *Rai1^+/−^* and 6 WT mice at 33–36 wks. All AUCs are computed until 120 minutes, for the entire length of the time curves.

To explore the contribution of *RAI1* copy number loss to the metabolic phenotypes of SMS, we also studied *Rai1* ^+/−^ mice [36], [37] on the same 129S5 (N>10) strain background as the *Df(11)17/+* mice. Similar to what we observed for *Df(11)17/+* mice, and in accordance with a previous study [18] conducted on a different genetic background, *Rai1* ^+/−^ males have both significantly increased body weight in adulthood (Figure 8A) and elevated overall proportion of body fat (Figure 8B). Also similar to the *Df(11)17/+* mice, *Rai1* ^+/−^ animals display an unchanged TC/HDL ratio, although they have both *elevated* TC and HDL levels, opposite to the reduced TC and HDL levels in *Df(11)17/+* mice (Figure 8C). Elevated cholesterol was also observed in *Rai1* ^+/−^ mice on C57BL/6J background in Burns *et al* [18], although the difference was not statistically significant, which may reflect the mixing of male and female mice and a potential dilution of the difference in male animals. Similarly, Burns *et al* also noted the increased proportion of body fat in both males and females, although the difference in their assay is only significant for the females. The differences in our findings for the male mice may result from different experimental approaches; ECHO-MRI is less subject to variations introduced by the individual experimentalist and potentially more objectively detects subtle differences in body composition. In addition, the impairment of *Rai1* ^+/−^ in glucose clearance becomes significant at later time points during the GTT assay (Figure 8D); these animals also show higher plasma insulin at fasting and during the GTT (Figure 8E), although there is no significant difference in blood glucose during ITT (Figure 8F and 8G).

Overall, *Rai1* ^+/−^ mice are similar to *Df(11)17/+* mice in their increased body weight and total body fat percentage as well as hyperinsulinemia, impaired GTT and an unchanged TC/HDL ratio. Intriguingly, Edelman *et al* [16] observed a higher percentage of obesity in SMS patients with *RAI1* point mutations (66.7%) than those with 17p11.2 deletions (12.9%). Although the number of SMS patients due to *RAI1* point mutation in that report is small (n = 9), these data nevertheless support a significant role for *RAI1* copy number loss in the overall metabolic phenotype of SMS, and also suggest possible contributions from other genes/genetic elements in the SMS deletion interval or the deletion *per se* [25].

## Discussion

**Figure 9:**
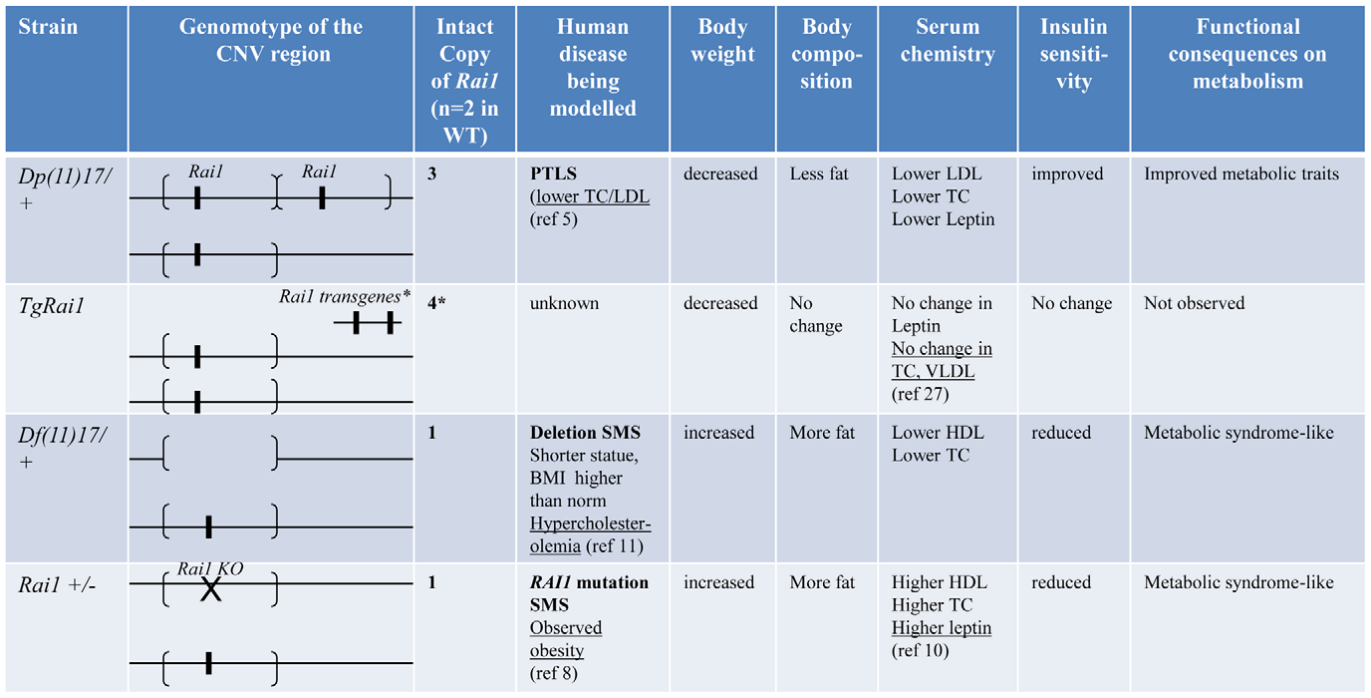
Experimental findings for specific genetic/genomic variations in this report. Genomotypes are shown similar to [57]. The segments flanked by brackets that encompass the *Rai1* gene represent the CNV region, duplicated in *Dp(11)17/+* or deleted in *Df(11)17/+*. *: *TgRai1* strain has the insertion of *Rai1* outside of chromosome 11; it gives similar *Rai1* expression levels to those of the *Dp(11)17/+* strain although the copy number has been determined as four [35]. Underlined results are from published reports.

In summary (Figure 9), our detailed analyses of mouse models and human patients demonstrate that the duplication CNV of PTLS conveys highly penetrant metabolic consequences that are antithetical to or “mirror” [9] those observed in MetS. At the same time, it confers protection against the development of diet induced obesity and insulin resistance. These phenotypes are not manifest in the transgenic *TgRai1* mice with a similarly increased level of *Rai1* expression but without the duplication CNV. In contrast, the reciprocal deletion CNV causes phenotypes that are opposite to those observed with the duplication CNV and that resemble a *bona fide* metabolic syndrome (summarized in Figure 9).

The reciprocal/mirror phenotypes caused by the reciprocal CNV of *Dp(11)17* and *Df(11)17* is interesting. Reciprocal phenotypes associated with opposing gene/genome dosage alterations (*i.e.* copy number loss versus copy number gain) have been described for the complex neuropsychiatric traits of schizophrenia and autism, as well as microcephaly and macrocephaly, associated with, respectively, duplication/deletion CNV at 16p11.2 [38], [39], [40] and deletion/duplication of 1q21.1 [41], [42], [43], [44]. Indeed, another pair of weight regulation associated duplication/deletion CNVs at 16p11.2 was also related to reciprocal changes in BMI and manifest leanness/obesity [9], [10], [27]. These reciprocal traits with opposing dosage alterations are consistent with the model of diametrically opposing phenotypes for genomic sister disorders postulated by Crespi *et al* [40], [45], [46].

Importantly, neither the duplication nor the deletion CNV associated phenotypes we describe herein in both human patients and mouse models can be attributed solely to the *RAI1/Rai1* gene, although *RAI1/Rai1* dosage loss does appear to partially contribute to the deletion phenotypes.

Besides *Rai1*, another gene *Srebf1* (coding for sterol regulatory element binding protein 1, Srebp1), that maps directly adjacent to *RAI1/Rai1* in both human/mouse genome and functions as a key regulator in the biosynthesis of fatty acid and cholesterol, is the only gene in the SMS/PTLS interval known to be involved in energy metabolism. Overexpression of the active N-terminal portion of Srebp1 protein does not change the plasma lipid profile [47] and results in mild insulin resistance [48]. Both phenotypes are distinctly different from the metabolism phenotype we observed in *Dp(11)17/+* mice, rendering the copy number gain of *Srebf1* unlikely the reason for the *Dp(11)17/+* metabolic phenotypes. Heterozygous *Srebf1* knockout mice *Srebf1^+/−^* were described as “phenotypically normal” [49]. *Srebf1^−/−^* animals are 50–85% embryonic lethal, but the surviving mice display unchanged body weight and slightly reduced total cholesterol and triglycerides in plasma [49]. The copy number loss of *Srebf1* in *Df(11)17/+* mice is thus also unlikely a major contributor to the observed metabolic phenotypes.

Recently, a microRNA miR33b was found to be embedded in an intron of human *SREBF1* [50], [51]. Together with its paralogue miR33a (embedded in the paralogue of *SREBF1*, *SREBF2*), miR33b targets the adenosine triphosphate-binding cassette transporter (ABCA1), decreases plasma HDL and boosts intracellular cholesterol levels in cooperation with SREBP proteins [50], [51], [52]. However, mouse *Srebf1* does not contain mir33b [50], [51]; it is thus not a candidate accounting for the metabolic phenotype observed in *Dp(11)17/+* and *Df(11)17/+* mice. The potential role of miR33b in human PTLS/SMS metabolic manifestation will have to be studied with different models, such as those that introduce a human miR33b into the mouse genome.

Our current knowledge thus does not support a “single gene” contribution of the dosage change of *Rai1*, *Srebf1* or any other known genetic element to the metabolic phenotypes of SMS/PTLS. However, it is distinctly possible that the copy number change of *RAI1* and *SREBF1* in *cis*, with one of them exerting an epistatic effect on the other or functioning as a modifier, is required for manifestation of the complete metabolic phenotype of PTLS/SMS. Further, the potential “*cis*” effect could also possibly involve other genetic elements in addition to *RAI1* and *SREBF1*. These metabolic manifestations would then belong to the category of “contiguous gene syndromes” [53] or genomic disorders [54], [55] that require multiple genes/genetic/genomic factors to work in concert, a concept referred to as *cis*-genetics and in contrast to the *trans* interactions of alleles at one locus formalized by Mendelism [56]. Similar mechanisms have been proposed for the craniofacial phenotypes of the SMS/*Df(11)17/+* mice, wherein the phenotypic penetrance is clearly modified by other genetic elements in the deletion interval although the copy loss of *Rai1* appears to be responsible for most of the traits [21], [22].

Further, a number of other mechanisms, including gene interruption and gene fusion due to CNV breakpoints, position effect, the unmasking of a recessive allele by a deletion, as well as potential effects of transvection can contribute to the functional consequence of a CNV [57]. None of them can be displayed by single nucleotide variations (SNV). Recently, it has been demonstrated experimentally that a genomic structural change *per se*, as in a large CNV, can cause altered expression and functional perturbation of other loci/genes localized to the same chromosome, but outside of the CNV [25]. All these mechanisms can potentially contribute to the salient effect of the *Dp(11)17* CNV on weight regulation and energy metabolism that does not appear to be attributed to the dosage change of any single gene or genetic element(s).

Overall, we show that a duplication CNV can result in a lean body phenotype, metabolic phenotypes in mirror image contrast to those observed in metabolic syndrome, and protect from diet induced obesity. Moreover, we demonstrate that these phenotypes are fully penetrant, independent of genetic background and resistant to environmental influences. Furthermore, we provide evidence that the CNV effects are due to more than dosage alteration of a single gene, a finding that highlights distinct functional significance of CNV as compared to SNVs. These findings confirm that CNVs can be causative for weight regulation and energy metabolism phenotypes and suggest that CNVs could play a major role in the common complex diseases of human obesity.

## Materials and Methods

### Animals

All animal studies were approved by Baylor College of Medicine IRB and carried out in accordance with Baylor IACUC. Mice were housed 2–5 per cage in a 12-hour light/12-hour dark cycle with access to food and water *ad libitum*.

### Body composition and Serum analyses

Body composition was analyzed with the ECHO-MRI system (Echo medical systems, Texas). Mouse serum was prepared from blood obtained through cardiac puncture and analyzed with the COBAS Integra 400 plus analyzer (Roche). Plasma leptin, FFA, adiponectin and glycerol levels were measured by using a Mouse Leptin ELISA Kit (Millipore), NEFA C Test Kit (Wako), Mouse Adiponectin ELISA Kit (Millipore) and Serum/plasma Glycerol detection kit (Sigma), respectively.

### Histology

Epididymal (EWAT), mesenteric (MWAT), retroperitoneal (RWAT) and inguinal (IWAT) white adipose tissues, as well as brown adipose tissues (BAT) and liver were dissected from mice deeply anesthetized with Isoflorane (Butler). Tissues were weighed and fixed in 4% neutral buffered formaldehyde (Fisher). Paraffin-embedded sections were stained with hematoxylin and eosin. Photomicrographs were captured by optic microscopy (Zeiss Axiostar Plus).

### Activity and metabolic rate measurements

The locomotion activity assay was performed in home cages by using the VersaMax Animal Activity Monitoring System (AccuScan Instruments). Mice were acclimated in the monitoring environments for at least 24 hours before the experiment.

Energy expenditure was measured using the CLAMS System (Columbus Instruments). Animals were allowed to acclimatize in the chambers for 72 hours, and measurements were taken subsequently for 72 hr during the light cycle and dark cycle while mice were freely allowed to access food and water. Oxygen consumption was normalized to lean tissue mass.

### Glucose Tolerance Test (GTT) and Insulin Tolerance Test (ITT)

For intraperitoneal GTT, 1.5 g of glucose/kg of body weight was injected after a 6-h fasting period. For ITT, an intraperitoneal injection of regular insulin (Humulin R; 1 unit/kg of body weight) was administered after a 4–6 h fasting. Blood glucose levels were measured using a glucometer (Life Scan).

### Protein extraction, immunoblotting, and quantitative RT–PCR

Tissues were lysed in RIPA buffer with Complete Protease Inhibitor Cocktail (Roche). Protein concentration was determined with BCA protein assay kit (Pierce); each sample was separated by SDS-PAGE and electro-transferred to nitrocellulose membrane for immunoblot analyses. Western blots for UCP1 protein were performed with antibody AB3036 (Millipore), after which the same blot was normalized to actin using MAB1501 (Millipore). The ImmunoCruz Western Blotting Luminol reagent (SantaCruz) was used as the substrate.

RNA was isolated with Trizol (Invitrogen), cDNA synthesized with SuperScript III System (Invitrogen), and RT-PCR was performed on the Strategene MX3000 real time detection system using iQ SYBR Green PCR reagent kit (Biorad).

### Statistical methods

Results are expressed as mean ± s.e.m. Comparisons between two groups were made using either two-tailed Student's *t*-test (EXCEL) or ANOVA repeated measures (SPSS), as appropriate. AUC analysis was performed using SigmaBlot. *P*<0.05 was considered to be statistically significant.

## Supporting Information

Figure S1 Mouse models of SMS and PTLS. The region on mouse chromosome 11 syntenic to the human the SMS/PTLS region on human chromosome 17 (synteny is indicated by gray shaded regions). Key genes that demarcate the SMS/PTLS region are shown. The thick black horizontal line above denotes the region of the SMS/PTLS common deletion/duplication. Shown below is the region deleted/duplicated in *Df(11)17/Dp(11)17* mice (bold horizontal bar with vertical bars on the end); asterisk * represents the knock-out mouse model of *Rai1*.
(PDF)

Figure S2 *TgRai1* mice (pink) have similar body composition (A), similar serum leptin level (B), and display similar glucose and insulin levels during IP-GTT (C, D) compared to WT mice (gray). All comparisons were made with two-tailed t-test; results are expressed as mean ± s.e.m. from measurements of (A) 9 *TgRai1* and 8 WT at 32–35 wks, via dissection of intra-abdominal fat pads (gonadal, retroperitoneal and mesenteric) and subcutaneous fat pads (dorsal, inguinal and groin) as a measure of total fat [18]. (B) 11 *TgRai1* and 10 WT at 10–20 wks by leptin ELISA assay at the University of Cincinnati Mouse Metabolic Phenotyping Core per standard protocols and (C, D) 6 *TgRai1* and 4 WT animals at 30–32 wks.
(PDF)

Figure S3 Z-scores for height_for_age (A, D, G), weight_for_age (B, E, H) and BMI_for_age (C, F, I) of SMS subjects are plotted as two way scatter plots (A to F) for male (black) (A, B, C) and female (gray) (D, E, F) separately and summarized as mean ± s.e.m. (G to I) with both genders. Subjects are grouped into group 1: 0–23 months; group 2: 2–5 years; group 3: 6–11 years; group 4: 12–19 years; group 5: ≥20years; asterisk (*): significant differences with comparison to the population normative values as calculated with 2-tailed one-sample t-test. All age groups from both genders differ from population norm in their height: for males, p<0.001 (group 1, 2), p = 0.002, 0.059 (#) and 0.006 for group 3 to 5; for females, p = 0.034 and 0.019 for group 1 and 5, p<0.001 for group 2 to 4. The weight of female group 1–3 differs from population norm: p<0.001 for group 1 and 2; p = 0.004 for group 3. Significantly higher BMI was found for male age group 2 to 4 (p = 0.04, 0.022, 0.007, 0.001) and female group 4 (p = 0.002).
(PDF)

Figure S4 Comparative expression analyses of (A, B) some signature metabolic genes in *Dp(11)17/+* mice (red) and (C, D) UCP1 in *Df(11)17/+* mice (green). (A, B) Relative mRNA abundance for a group of signature genes for energy metabolism: (A) *AP2*, *Ucp1*, *Ucp2*, *Ucp3* and *Glut4* (B) *Lpk*, *Fas*, *Acc1*, *Srebp1c*, *Tnxip* was determined in BAT (A) and liver (B) of *Dp(11)17/+* and WT mice. None of the genes displayed significant expression difference between two genotypes: (A) *Ap2* (p = 0.79), *Ucp1* (p = 0.27), *Ucp2* (p = 0.12), *Ucp3* (p = 0.08) *Glut4* (p = 0.83). (B) *Lpk* (p = 0.65), *Fas* (p = 0.15), *Acc1* (p = 0.94), *Srebp1c* (p = 0.75), *Tnxip* (p = 0.62). (C) Western blot for UCP1 expression in BAT tissue of four *Df(11)17/+* and four WT mice with antibody AB3036 (Millipore) and normalized to actin blotting using MAB1501 (Millipore). (D) Normalized intensity of UCP1 signals in *Df(11)17/+* vs. WT mice (1.776±0.154 vs. 1.186±0.059, p = 0.154). The measurements are from (A, B) 3 *Dp(11)17*/+ and 3 WT at 30 wks. (C, D) 4 *Df(11)17*/+ and 4 WT at 30 wks.
(PDF)

Table S1 Serum chemistry comparison of *Dp(11)17/+* and WT mice. Results are expressed as mean ± s.e.m. and are calculated from the measurements of 5 *Dp(11)17/+* and 6 WT males at 21–22 wks. For clarity, the measurements, but not the p-values are shown in bold.
(PDF)
