# Supplementary figures and images for: A Duplication CNV That Conveys Traits Reciprocal to Metabolic Syndrome and Protects against Diet-Induced Obesity in Mice and Men
Source: PLoS Genet. 2012 May 24;8(5):e1002713. doi: 10.1371/journal.pgen.1002713 (PMC3359973; doi:10.1371/journal.pgen.1002713)

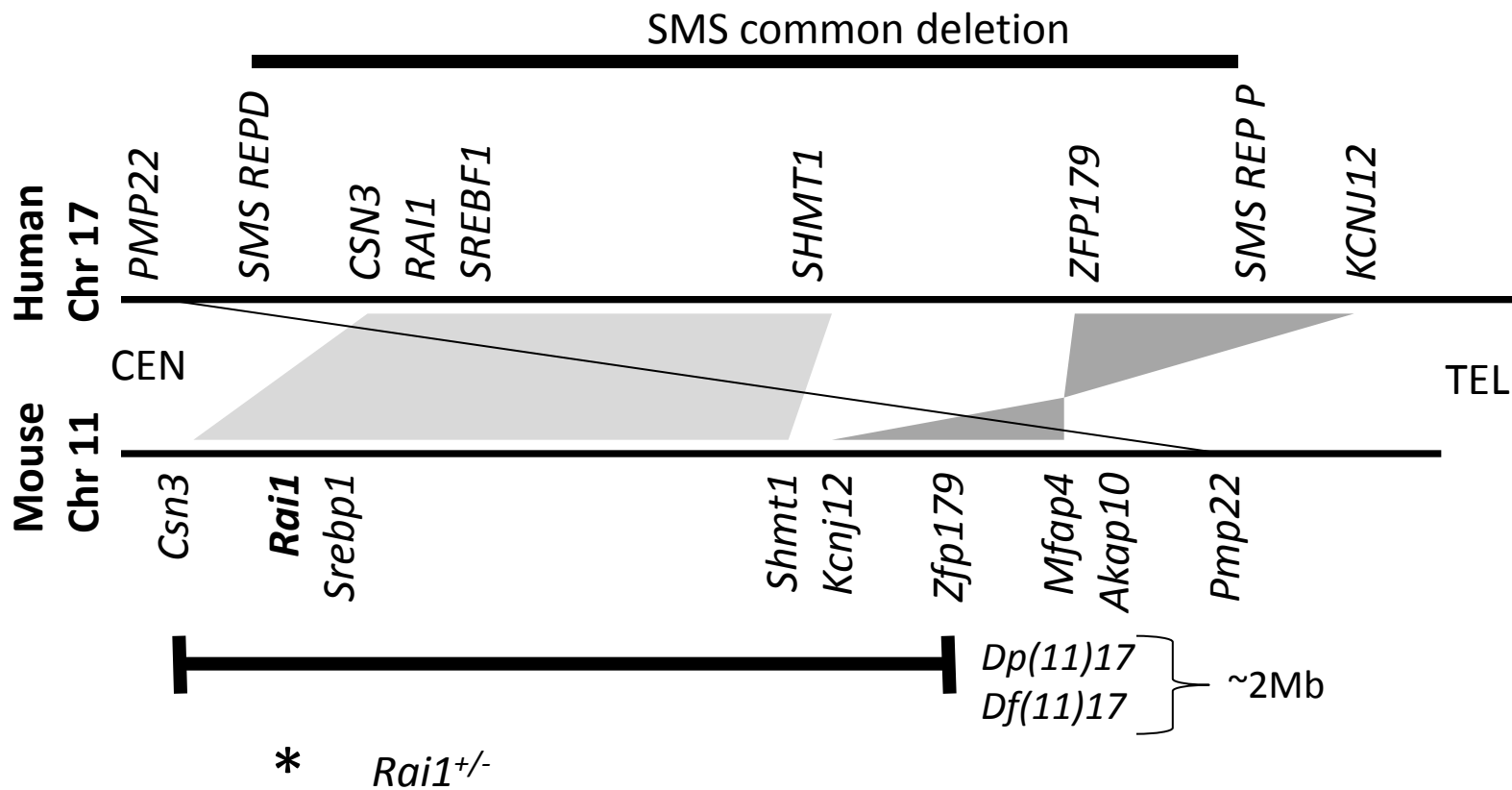

Supplement: Figure S1 — Mouse models of SMS and PTLS. The region on mouse chromosome 11 syntenic to the human the SMS/PTLS region on human chromosome 17 (synteny is indicated by gray shaded regions). Key genes that demarcate the SMS/PTLS region are shown. The thick black horizontal line above denotes the region of the SMS/PTLS common deletion/duplication. Shown below is the region deleted/duplicated in Df(11)17/Dp(11)17 mice (bold horizontal bar with vertical bars on the end); asterisk * represents the knock-out mouse model of Rai1. (PDF) [file pgen.1002713.s001.pdf]

**A**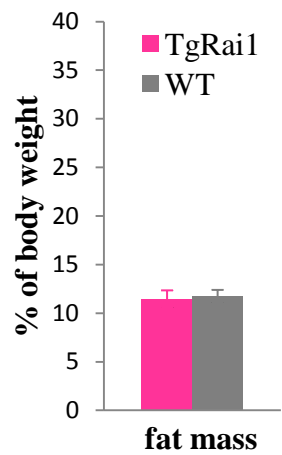**B**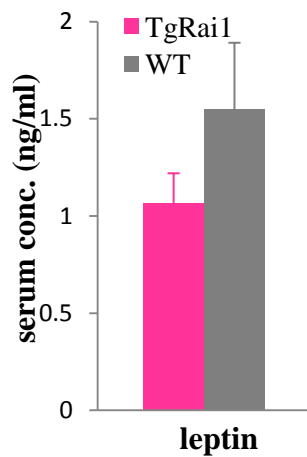**C**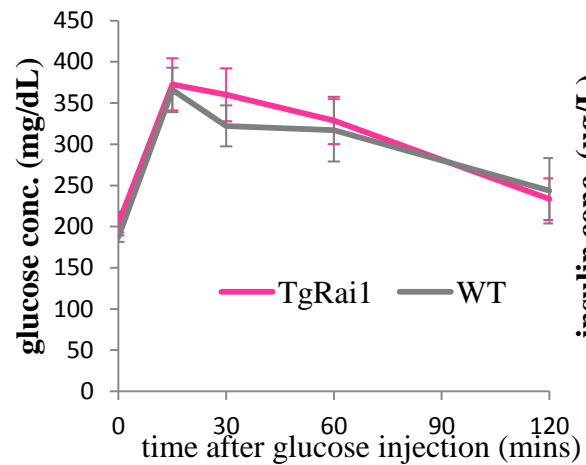**D**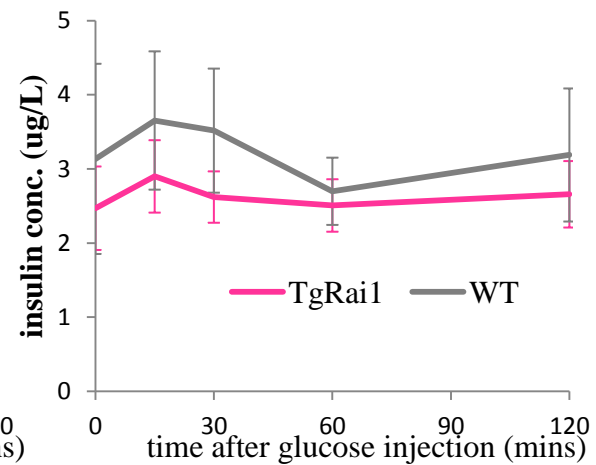

Supplement: Figure S2 — TgRai1 mice (pink) have similar body composition (A), similar serum leptin level (B), and display similar glucose and insulin levels during IP-GTT (C, D) compared to WT mice (gray). All comparisons were made with two-tailed t-test; results are expressed as mean ± s.e.m. from measurements of (A) 9 TgRai1 and 8 WT at 32–35 wks, via dissection of intra-abdominal fat pads (gonadal, retroperitoneal and mesenteric) and subcutaneous fat pads (dorsal, inguinal and groin) as a measure of total fat [18]. (B) 11 TgRai1 and 10 WT at 10–20 wks by leptin ELISA assay at the University of Cincinnati Mouse Metabolic Phenotyping Core per standard protocols and (C, D) 6 TgRai1 and 4 WT animals at 30–32 wks. (PDF) [file pgen.1002713.s002.pdf]

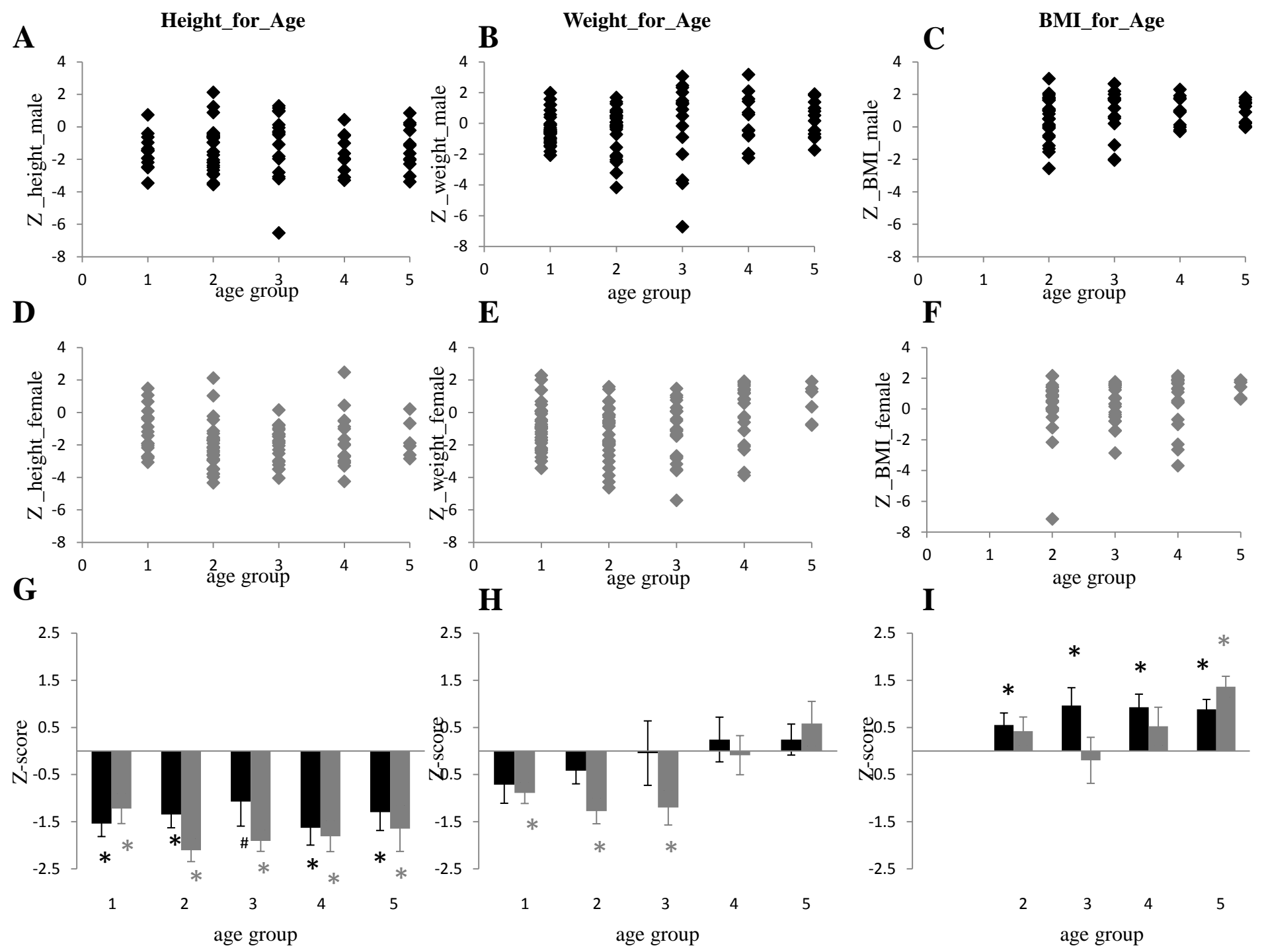

Supplement: Figure S3 — Z-scores for height_for_age (A, D, G), weight_for_age (B, E, H) and BMI_for_age (C, F, I) of SMS subjects are plotted as two way scatter plots (A to F) for male (black) (A, B, C) and female (gray) (D, E, F) separately and summarized as mean ± s.e.m. (G to I) with both genders. Subjects are grouped into group 1: 0–23 months; group 2: 2–5 years; group 3: 6–11 years; group 4: 12–19 years; group 5: ≥20years; asterisk (*): significant differences with comparison to the population normative values as calculated with 2-tailed one-sample t-test. All age groups from both genders differ from population norm in their height: for males, p<0.001 (group 1, 2), p = 0.002, 0.059 (#) and 0.006 for group 3 to 5; for females, p = 0.034 and 0.019 for group 1 and 5, p<0.001 for group 2 to 4. The weight of female group 1–3 differs from population norm: p<0.001 for group 1 and 2; p = 0.004 for group 3. Significantly higher BMI was found for male age group 2 to 4 (p = 0.04, 0.022, 0.007, 0.001) and female group 4 (p = 0.002). (PDF) [file pgen.1002713.s003.pdf]

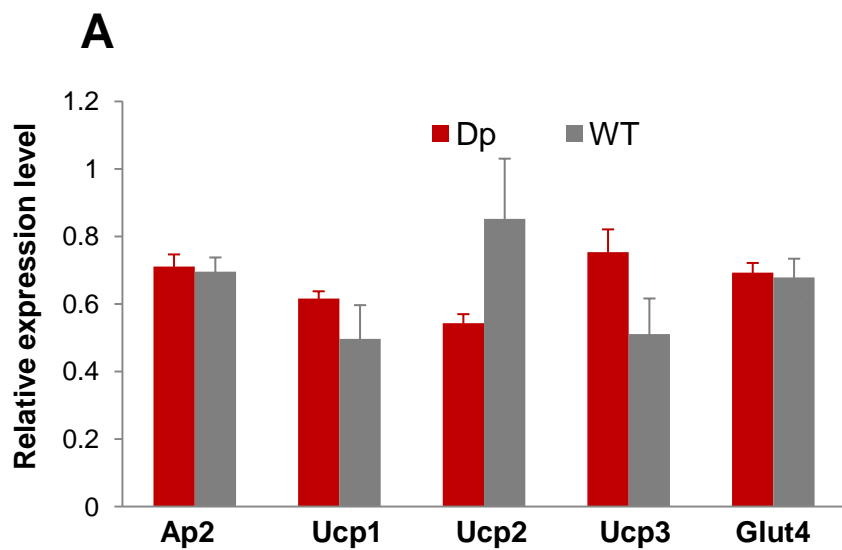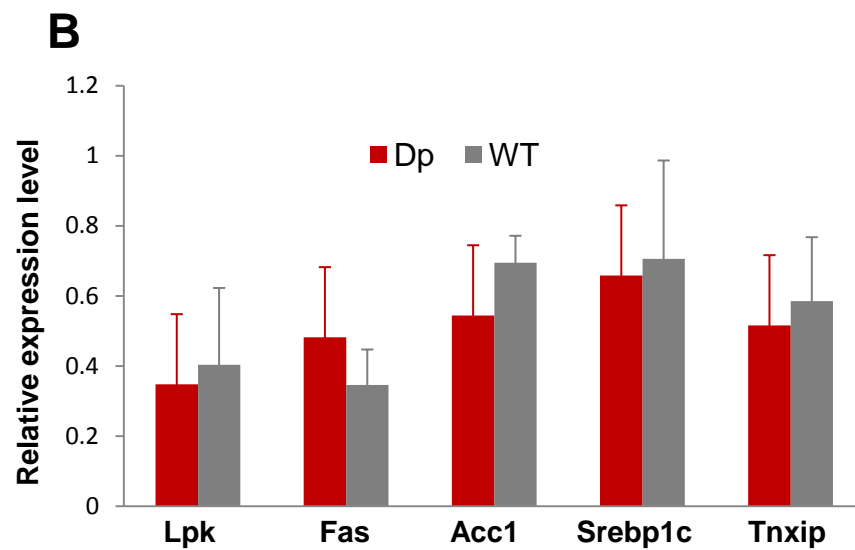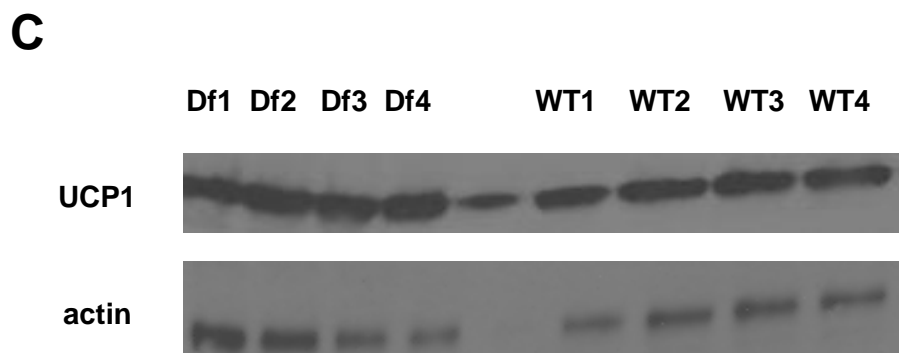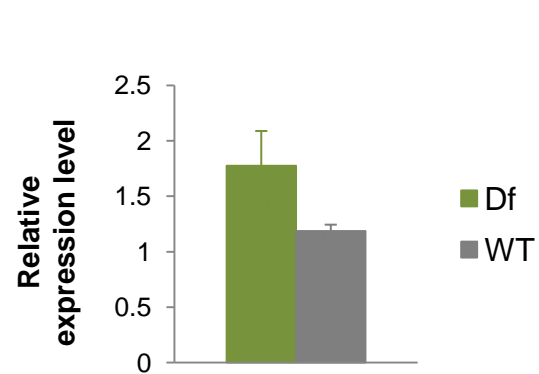

Supplement: Figure S4 — Comparative expression analyses of (A, B) some signature metabolic genes in Dp(11)17/+ mice (red) and (C, D) UCP1 in Df(11)17/+ mice (green). (A, B) Relative mRNA abundance for a group of signature genes for energy metabolism: (A) AP2, Ucp1, Ucp2, Ucp3 and Glut4 (B) Lpk, Fas, Acc1, Srebp1c, Tnxip was determined in BAT (A) and liver (B) of Dp(11)17/+ and WT mice. None of the genes displayed significant expression difference between two genotypes: (A) Ap2 (p = 0.79), Ucp1 (p = 0.27), Ucp2 (p = 0.12), Ucp3 (p = 0.08) Glut4 (p = 0.83). (B) Lpk (p = 0.65), Fas (p = 0.15), Acc1 (p = 0.94), Srebp1c (p = 0.75), Tnxip (p = 0.62). (C) Western blot for UCP1 expression in BAT tissue of four Df(11)17/+ and four WT mice with antibody AB3036 (Millipore) and normalized to actin blotting using MAB1501 (Millipore). (D) Normalized intensity of UCP1 signals in Df(11)17/+ vs. WT mice (1.776±0.154 vs. 1.186±0.059, p = 0.154). The measurements are from (A, B) 3 Dp(11)17/+ and 3 WT at 30 wks. (C, D) 4 Df(11)17/+ and 4 WT at 30 wks. (PDF) [file pgen.1002713.s004.pdf]
