# Supplementary material for: A Duplication CNV That Conveys Traits Reciprocal to Metabolic Syndrome and Protects against Diet-Induced Obesity in Mice and Men
Source: PLoS Genet. 2012 May 24;8(5):e1002713. doi: 10.1371/journal.pgen.1002713 (PMC3359973; doi:10.1371/journal.pgen.1002713)
Supplement: Table S1 — Serum chemistry comparison of Dp(11)17/+ and WT mice. Results are expressed as mean ± s.e.m. and are calculated from the measurements of 5 Dp(11)17/+ and 6 WT males at 21–22 wks. For clarity, the measurements, but not the p-values are shown in bold. (PDF) [file pgen.1002713.s005.pdf]

Suppl Table 1: Serum chemistry comparison of *Dp(11)17/+* and WT mice

	**CREA** mg/dL	**Total protein** g/dL	**A/G ratio**	**ALT** U/L	**AST** U/L
Dp(11)17 /+	**0.12±0.02**	**4.72±0.16**	**1.96±0.21**	**244±61.83**	**478±121.67**
**WT**	**0.1±0.00**	**5.45±0.29**	**1.88±0.03**	**79.83±18.35**	**230.5±47.08**
p value	0.373901	0.061238	0.736871	0.054534	0.114108
	**ALP** U/L	**CK** U/L	**LDH** U/L	**BUN** mg/dL	**Calcium** mg/dL
Dp(11)17 /+	**70±19.32**	**1125.4±875.61**	**492.8±260.02**	**20.1±1.19**	**8.94±0.29**
**WT**	**64.5±6.17**	**778.17±166.62**	**714±144.30**	**19.72±0.92**	**9.18±0.39**
p value	0.797513	0.715426	0.483535	0.804964	0.630496
	**Glucose** mg/dL	**Total Bilirubin** mg/dL	**direct Bilirubin** mg/dL	**indirect Bilirubin** mg/dL	**Magnesium** mg/dL
Dp(11)17 /+	**154±31.84**	**0.246±0.06**	**0.066±0.03**	**0.2±0.03**	**3.18±0.17**
**WT**	**198.67±19.03**	**0.25±0.01**	**0.028±0.01**	**0.23±0.02**	**3.35±0.12**
p value	0.269486	0.99163	0.314965	0.408738	0.434566
	**Phosphate** mg/dL				
Dp(11)17 /+	**9.16±1.10**				
**WT**	**7.85±0.39**				
p value	0.31187				

CREA: Creatinine
A/G ratio: Albumin/globulin ratio
ALT: Alanine transaminase
AST: Aspartate transaminase
ALP: Alkaline phosphatase
CK: Creatine kinase
LDH: Lactate dehydrogenase
BUN:blood urea nitrogen
Tt/di/indi Bilirubin: total/direct(conjugated)/indirect bilirubin
